# Development of Cyclodextrin-Based Mono and Dual Encapsulated Powders by Spray Drying for Successful Preservation of Everlasting Flower Extract

**DOI:** 10.3390/pharmaceutics16070861

**Published:** 2024-06-27

**Authors:** Nada Ćujić Nikolić, Miloš Jovanović, Milica Radan, Zorica Lazarević, Dubravka Bigović, Smilja Marković, Nataša Jovanović Lješković, Katarina Šavikin

**Affiliations:** 1 Institute for Medicinal Plants Research “Dr. Josif Pančić”, Tadeuša Košćuška 1, 11000 Belgrade, Serbia; mradan@mocbilja.rs (M.R.); zdrinic@mocbilja.rs (Z.L.); dbigovic@mocbilja.rs (D.B.); ksavikin@mocbilja.rs (K.Š.); 2 Faculty of Medicine, Department of Pharmacy, University of Niš, Boulevard Dr. Zorana Đinđića 81, 18000 Niš, Serbia; milos.jovanovic@medfak.ni.ac.rs; 3 Institute of Technical Sciences of SASA, Knez Mihailova 35/IV, 11000 Belgrade, Serbia; smarkovic@itn.sanu.ac.rs; 4 Faculty of Pharmacy Novi Sad, University of Business Academy, Trg Mladenaca 5, 21101 Novi Sad, Serbia; natasa.ljeskovic@faculty-pharmacy.com

**Keywords:** everlasting flowers, *Helichrysum plicatum* DC., spray drying, dual-microencapsulation, cyclodextrins, particle characterization, phenolics

## Abstract

The study aimed to develop encapsulation systems to maintain the preservation of everlasting (*Helichrysum plicatum*) flower extract polyphenols. Spray-dried encapsulates were formulated using β-cyclodextrin (BCD) and 2-hydroxypropyl-β-cyclodextrin (HPBCD) as supramolecular hosts, and their macromolecule mixtures with the conventional carriers, maltodextrin (MD) and whey protein (WP). The obtained microparticles were comparatively assessed regarding technological, physicochemical, and phytochemical properties. The highest yields were achieved by combining cyclodextrins with whey protein (73.96% for WP+BCD and 75.50% for WP+HPBCD compared to 62.48% of pure extract). The extract–carrier interactions and thermal stability were evaluated by FTIR and DSC analysis, suggesting successful entrapment within the carriers. Carriers reduced the particle diameter (3.99 to 4.86 μm compared to 6.49 μm of pure extract), classifying all encapsulates as microsystems. Carrier blends made the particle size distribution uniform, while SEM analysis revealed the production of more spherical and less aggregated particles. The HPBCD provided the highest encapsulation efficiency, with the highest content of detected aglycones and slightly lower values of their glycosylated forms. An analysis of the dual macromolecule encapsulation systems revealed the highest bioactive preservation potential for SHE+MD+BCD and SHE+WP+HPBCD. Overall, macromolecule combinations of cyclodextrins and conventional biopolymers in the spray-drying process can enhance the functional properties of *H. plicatum* extract.

## 1. Introduction

Everlasting flowers are the common name for plants with distinctive bright-yellow flowers belonging to the genus *Helichrysum* Miller (Asteraceae). The genus Helichrysum includes about 600 widely distributed plant species traditionally used for medicinal, cosmetic, and food purposes [1]. Researchers’ interest in this genus as a significant source of pharmacologically active secondary metabolites has increased recently. The most studied metabolites of *Helichrysum* species belong to pyrones, phloroglucinols, benzofurans, phthalides, flavonoids, chalcones, phenolic acids, coumarins, terpenes, and essential oils [1,2]. *Helichrysum plicatum* DC. is a member of this genus originating from the Balkan Peninsula, Anatolian Peninsula, and Iran [3]. Preparations of *H. plicatum* are traditionally used for gastrointestinal complaints such as abdominal pain, jaundice, liver disorders, colic, dysmenorrhea, dysuria, kidney stones, diabetes, rheumatism, epilepsy, and wound healing [1,2,4]. Most of these applications could be related to the proven spasmolytic, antioxidant, and antimicrobial activities of *H. plicatum* extracts [3,4,5]. The polar fractions of *Helichrysum* extracts consist of flavonoids, chalcones, and phenolic acids, which appear responsible for potent pharmacological activities [1]. The flavonoids naringenin, kaempferol, apigenin, and glycoside derivatives are the dominant polyphenolics of *H. plicatum* flower extract [3].

A critical issue in developing formulations containing polyphenolic bioactives is their limited stability with elevated temperatures, pH fluctuations, and exposure to oxygen, light, and moisture. These limiting factors can be resolved by coating the extract with polymer layers using various microencapsulation techniques. Indeed, the polymer layers create a microenvironment that protects the polyphenols from external degrading factors, thus improving their stability [6]. Spray drying is an extensively used technique for microencapsulating a wide range of bioactive compounds. The operating principle is to preserve a mixture of liquid extract (core material) and encapsulation agent (wall material) in a stream of heated air, entrapping the extract in one step and drying it into powdered microparticles. Spray drying is a cost-effective, versatile, continuous, and easily feasible method scalable on an industrial level. Spray-dried microcapsules allow easy handling, storage, and dosing into final formulations [7]. The selection of wall materials is one of the critical factors in the development of spray-dried microencapsulated systems. The selection of the type and concentration of the wall material can affect the technological properties of the microparticles such as encapsulation efficiency, bioavailability, and stability [6].

Carbohydrates and protein biopolymers are the most commonly used natural wall materials in spray drying [7,8,9,10]. Among them, maltodextrin is the most widely used as a cheap, tasteless, low-viscosity, and easily soluble excipient in commercial formulations [11]. On the other hand, the disadvantages of maltodextrin are its high glycemic index, pronounced hydrophilicity, and poor emulsification properties [6]. Accordingly, maltodextrin can be combined with other wall materials with complementary properties to obtain the desired coating film depending on the core materials and the desired technological performances of the microparticles.

Cyclodextrin-based biopolymers (CDs) are cyclic, toroidal-shaped, oligosaccharides composed of six, seven, or eight (α-CD, β-CD, or γ-CD, respectively) D-glucoside units linked by α (1,4) glycosidic bonds. These amphiphilic molecules owe their unique structure to a polar exterior surface and a hydrophobic inner cavity, generating an inclusion complex with guest bioactives [12]. CDs form inclusion complexes with the compounds, improving their solubility, stability, and bioavailability [7,13]. Including the bioactive compounds in the internal cavity causes their modified release, making these systems suitable for sustained-release formulations [12,14]. Natural cyclodextrins are listed by the US FDA as generally regarded as safe for utilization as food additives [15]. Modified CDs such as 2-hydroxypropyl-β-cyclodextrins (HPBCD) show better complexation and solubility properties compared to native β-cyclodextrins (BCD) [14]. Moreover, CDs themselves are useful for body weight control due to their low glycemic index and prebiotic features [16]. These biopolymers also represent great promise as an innovative strategy for effective therapies with minimal side effects [17,18].

Whey protein (WP) is a nutritionally and functionally valuable waste from the cheesemaking industry. WP possesses excellent gelling and emulsifying properties and therefore, represents a good wall material for encapsulation. Beyond this, cross-linked WP macromolecule components (β-lactoglobulin, α-lactalbumin, immunoglobulins, and serum albumin) can prove to have superior encapsulation efficiency and protection for sensitive compounds [7]. According to the literature of the author Jovanović et al., 2021, WP can be more effective in encapsulating some polyphenolic compounds compared to maltodextrin [19]. One of the disadvantages of WP is that the final spray-dried particles are irregularly shaped, rough, and agglomerated [20]. An improvement of drying properties and reduction of wall damage can be achieved by incorporating some other supports as secondary carriers. This improves the formation of a dry crust that surrounds the atomized droplets, thus reducing the oxidative degradation of the core materials. Particles with WP can also be used in the formulations with a modified release of the active ingredients [7].

This study hypothesized that a macromolecule combination of conventional and novel wall materials represents a promising approach for the dual encapsulation of bioactive compounds from medicinal plants, to obtain preserved micro-sized powdered forms that could open up a new perspective for the food and pharmaceutical industries. To the best of our knowledge, there are no studies of the microencapsulation process by the spray drying technique of any *Helichrysum* species with the addition of conventional wall material and novel material in combination. In light of this, this study aimed to pioneer the microencapsulation of everlasting (*H. plicatum*) flower extract as a feasible process using β-cyclodextrin, 2-hydroxypropyl-β-cyclodextrin, maltodextrin, and whey protein, alone and in combinations as wall materials. A unique concept of biopolymer combinations was established to preserve highly potent bioactives of everlasting flower extract, following the principles of the green and sustainable encapsulation technique, obtaining tailor-made microencapsulated systems, with improved biopharmaceutical properties. Through comprehensive evaluation encompassing technological and physicochemical, along with phytochemical analyses, key insights that could significantly enhance the protection of the extract’s active compounds were achieved. The proposed microencapsulated powders have huge potential and great perspectives for application as highly functional, sophisticated phytopharmaceutical products.

## 2. Materials and Method

### 2.1. Chemicals

Maltodextrin (MD) (DE_16–19.9_) was provided by Davisco Foods International (Le Sueur, MN, USA), whey protein (WP) was provided by Polmlek (Raciąż, Poland), and *β*-cyclodextrin (BCD) (98% grade) and 2-hydroxypropyl-*β*-cyclodextrin (HPBCD) (97% grade) were provided by Acros Organics (Geel, Belgium). Folin–Ciocalteu reagent, gallic acid, orthophosphoric acid, and sodium carbonate were purchased from Sigma-Aldrich Chemie GmbH (Munich, Germany). Ultrapure water was prepared using a Milli-Q purification system (Millipore, France), and HPLC-grade acetonitrile was obtained from Merck (Darmstadt, Germany). Standards including kaempferol, apigenin, naringenin, isoquercitrin, kaempferol-3-*O*-glucoside, and apigenin-7-*O*-glucoside were provided from Extrasynthese (Cedex, Genay, France).

### 2.2. Plant Material and Preparation of Extract

The dried everlasting (*H. plicatum*) flowers were purchased from the Institute for Medicinal Plants Research “Dr. Josif Pančić” (Belgrade, Serbia). The plant material was crushed in a laboratory mill and subjected to a percolation process carried out under the conditions previously published by Bigović et al. [4]. An ethanol–water mixture (50:50) was used for 12 h of the extraction process, whereas the solid-to-solvent ratio was 1:5. After the percolation process, the ethanol was evaporated under vacuum by a rotary evaporator (Buchi rotavapor R-114, Flawil, Swizerland), at 50 °C. The obtained liquid *H. plicatum* extract (LHE) was collected and used for future experiments.

### 2.3. Spray-Drying Process

The prepared LHE was spray-dried with and without the carrier addition. Four different biopolymers were used: MD and WP in concentrations of 20%, *w*/*w* [20] and BCD and HPBCD in 15%, *w*/*w* [21,22]. The carrier concentrations used in the experiments were calculated according to the dry weight of LHE. Each biopolymer was dissolved separately in a previously prepared LHE, while the cyclodextrin solutions were prepared 24 h before the spray-drying process, to perform a micellization process [23]. For the carrier combinations, MD and WP were added to the CD solutions after the micellization process. Before spray drying, the prepared solutions were heated to 40 °C and mixed using a magnetic stirrer until complete homogenization. The starting liquid feed was spray-dried in a Labtex ESDTi spray dryer (Labtex, Huddersfield, UK) with a 0.5 mm standard diameter nozzle under the following conditions: inlet temperature 130 ± 5 °C, outlet temperature 70 ± 5 °C, spraying air flow rate (75 m^3^/h), liquid feed (11 mL/min rate), and atomization pressure (2 bar). Experimental drying conditions such as inlet and outlet temperature, flow rate, and the rate of liquid feed were fixed during the experiments. Due to the different carriers used, with wide viscosity ranges, one set of spray-drying operating conditions needed to be selected to enable the comparison of the product yield, encapsulation efficiency, and other parameters of each sample.

The obtained spray-dried *H. plicatum* extract (SHE) and SHE with carrier combinations were separated from the air by a cyclone. Nine free-flowing powders were obtained and transferred to high-density glass bottles before analyses. They were stored in the dark, in a desiccator at room temperature, and these conditions ensured physical stability and active compound preservation.

Nine powders prepared by the spray-drying method were characterized on technological properties (powder yield, moisture content, powder densities, flowability, cohesiveness, hygroscopicity, rehydration time, and pH), along with microparticles characterization of their physicochemical properties (particle size distribution, thermal stability, and the FTIR analysis) and chemical composition (the analysis of total and individual compounds).

### 2.4. Technological Characterization of Spray-Dried Powders

#### 2.4.1. Powder Yield

Drying process yield (Y) was calculated as the ratio between SHE masses (g) and the expected mass after the drying process:

Y (%) = m_extract_/m_expected_ × 100 (1)

Expected mass was calculated as the sum of dry residue in LHE multiplied by the mass of LHE used for the drying process and the mass of the carrier used:

m_expected_ (g) = m_carrier_ + m_dry residue_ × m_LHE_ (2)

#### 2.4.2. Moisture Content

The moisture content of the obtained samples was determined gravimetrically using a Halogen Moisture Analyzer HB43-S (Mettler Toledo, Greifensee, Switzerland). All samples were dried at 105 °C until a constant weight, and moisture content was calculated from the difference in mass before and after drying. All measurements were made in triplicate and the results are expressed as percentages (%, *w*/*w*).

#### 2.4.3. Bulk Density

Bulk densities of microencapsulates were examined according to the method by Vidović et al. [24], with slight modifications. One gram of each powder was deposed into a 5 mL graduated glass cylinder. The glass cylinder was agitated at 300 rpm on a shaker for 5 min (Unimax 1010, Heidolph, Schwabach, Germany), at ambient temperature. After the vibration process, volumes of dried powders were measured directly in a glass cylinder. Bulk densities were calculated as the powder mass ratios and volumes, and were expressed as milligrams of dried powder per milliliter (mg/mL). Therefore, the cylinder was tapped 120 times and the sample volumes were measured to determine the tapped densities [25]. Flowability and cohesiveness values of the samples were determined as Carr index (CI) and Hausner ratio (HR), respectively, and calculated using these Equations:

CI = (*ƍ*_tapped_ − *ƍ*_bulk_)/*ƍ*_tapped_ × 100 (3)

HR = *ƍ*_tapped_/*ƍ*_bulk_, (4)

where *ƍ*_tapped_ and *ƍ*_bulk_ represent tapped and bulk densities, respectively.

#### 2.4.4. Hygroscopicity

The powder’s hygroscopicity was determined according to the method of Cai & Corke [26], also described by M. Jovanović, Ćujić-Nikolić, et al. [11], with a slight moderation of monitored days. Roughly, 1 g of samples was positioned in a stability chamber, in conditions at ambient temperature (Memmert, Schwabach, Germany), with the addition of NaCl saturated solution (70% RH). Hygroscopicity was observed for 6 days (days 1, 3, 4, and 6). Results were expressed in percent (%), and calculated as grams of absorbed water (moisture) per 100 g of powders (g/100 g).

#### 2.4.5. Rehydration Time and pH Values

Rehydration time is considered the period for the complete dissolution of the obtained powders in water as a medium, at room temperature. Tests were carried out on a magnetic stirrer, and the period for the full reconstitution of 1 g of powder in 50 mL of water was measured, expressed in seconds (s) [27].

In each examined sample, pH values have been determined using Hanna instruments HI 99161 (Woonsocket, RI, USA).

### 2.5. Physicochemical Characterization of Spray-Dried Powders

#### 2.5.1. Particle Size Distribution

The particle size distribution for each dried powder was determined and quantified using a Mastersizer 2000 analyzer (Malvern Instruments, Worcestershire, UK). The d_10_, d_50_, and d_90_ parameters were determined, representing the sizes in µm of the 10%, 50%, and 90% of particles that are smaller than the remaining particles. The indicator of the particle size distribution (PSD) width was defined through the span value, calculated as (d_90_ − d_10_)/d_50_. D [3,2] as the surface-weighted mean, D [4,3] as the volume-weighted mean, and the uniformity of microparticles was determined as well.

#### 2.5.2. Microparticles Composition Analysis by FTIR Spectroscopy

Fourier-transform infrared (FTIR) spectra of the obtained powders (spray-dried SHE, spray-dried SHE with carriers, and pure carriers) were observed in the range mode between 400 and 4000 cm^−1^ using a Nicolet iS10 (Thermo Scientific, Stockholm, Sweden) spectrometer.

#### 2.5.3. Microparticles Morphology Determination by SEM Analysis

Scanning electron microscopy (SEM) analyses of the examined powder samples were performed using a JEOL JSM 6390LV scanning electron microscope (JEOL, Tokyo, Japan). Powders were attached with double-sided adhesive tape to SEM stubs, coated with 50 nm of gold layer, and treated for 100 s and under the 30 mA ion (Sputter Coater BAL-TEC SCD 005, Leica Microsystems, Wetzlar, Germany). All examined samples were observed at a magnification of ×1000, 7000, and 15,000, where all structures were clearly defined. 

#### 2.5.4. Microparticles Thermal Characterization by Differential Scanning Calorimetry (DSC)

The thermal properties of spray-dried powders were analyzed by DSC131 Evo (SETARAM Instrumentation, Caluire-et-Cuire, France). The samples were positioned in aluminum pans (30 µL), which were afterward hermetically sealed. An empty pan was used as a blind probe. The heating process was set and then both pans (reference and sample) were stabilized at 20 °C for 5 min, then heated to 200 °C, with a heating rate of 10 °C/min. The nitrogen flow was 20 mL/min. A baseline run was performed using empty pans under the same conditions, whereas the baseline subtraction and determination of enthalpy (J/g) were carried out by CALISTO PROCESSING software version 1.38 equipped with SETARAM Instrumentation (Caluire-et-Cuire, France).

### 2.6. Chemical Characterization of Microparticles

#### 2.6.1. Analysis of Total Phenolic Content (TPC)

For total polyphenols determination, a Folin–Ciocalteu assay with slight modifications was applied [28]. Approximately 7.5 mg of SHEs were dissolved in 10 mL of distilled water. The 200 μL of each sample with the addition of 1000 μL of 10% Folin–Ciocalteu reagent and, after four minutes, 800 μL of 7.5% Na_2_CO_3_ made the reaction mixture. A blank control contained distilled water instead of a sample in the reaction mixture. The samples were incubated in the dark for 2 h and after that absorbance was measured at 765 nm. The obtained results were presented as milligrams of gallic acid equivalent per gram of spray-dried powder (mg GAE/g).

#### 2.6.2. Analysis of Total Flavonoid Content (TFC)

The total flavonoid content of the examined powders was determined spectrophotometrically by a method based on the formation of a flavonoid–aluminum complex, according to Loizzo et al., with slight modifications [29]. Amounts around 37.5 mg of SHEs were dissolved in 10 mL of distilled water. The reaction mixtures were prepared with 200 µL of sample solution, and 800 µL of distilled water with the addition of 60 µL of 5% (*w*/*v*) sodium nitrite, which was mixed in a 2 mL volume eppendorf. After 5 min, 60 µL of 10% (*w*/*v*) AlCl_3_ was added to the mixture solution, followed by 400 µL of 1 M NaOH at 6 min, and the addition of distilled water to 2 mL. After these reactions, absorbance was measured at 510 nm. Total flavonoid content was determined in triplicate and expressed as catechin equivalents in mg/g of dried samples.

#### 2.6.3. Analysis of Individual Compounds in Microencapsulates by HPLC Method

The liquid *H. plicatum* flower extract and microencapsulated powders were analyzed using a High-Performance Liquid Chromatography (HPLC) (Аgilent Technologies 1260 Series, Waldbronn, Germany) system equipped with a DAD detector. The chromatographic separation was carried out on a Lithosphere RP-18 (250 × 4.0 mm, 5 μm) HPLC column under a water/1% orthophosphoric acid (A)–acetonitrile (B) gradient elution as follows: 0–5 min, 90–80% A; 5–10 min, 80% A; 10–20 min, 80–70% A; 20–30 min, 70–30% A; and 30–35 min, 30–0% A, based on the previously described method with some modifications [30]. The solvent flow rate was set to 1 mL/min, and the volume of injection was 10 µL. Individual ingredients were identified according to the retention times of the authentic standards, while the quantification was achieved by the calibration curve method (R^2^ > 0.99). The wavelength of the detector was set at 270 nm (for isoquercitrin, kaempferol-3-*O*-glucoside, kaempferol, and naringenin and its derivatives) and 340 nm (for apigenin-7-*O*-glucoside and apigenin). Results were expressed as milligrams per gram of dry weight of the liquid extract or powders (mg/g DW).

### 2.7. Encapsulation Efficiency of Bioactive Compounds in SHE Microencapsulates

The encapsulation efficiency (EE%) for all microencapsulated powders was calculated according to the following equation:

EE (%) = E/E_total_ × 100, (5)

where E represents the quantities of total polyphenols microencapsulated in the powders, and E_total_ presents the quantities of total polyphenols and their respective amount in the LHE.

### 2.8. Statistical Analysis

All experiments were performed in triplicate determinations and results were estimated as mean value ± standard deviation. Statistical analysis was performed using MS Office Excel v. 2010. One-way ANOVA was conducted to test the influence of individual factors on observed properties. The Duncan post hoc test was used for the estimation of the differences between the detection of the mean value. Significant levels were considered at *p* ≤ 0.05 (STATISTICA v.7.0.3). Principal component analysis (PCA) was performed using Origin Pro 2023 software (OriginLab Corporation, Northampton, MA, USA).

## 3. Results and Discussion

### 3.1. Technological Characterization of the Spray-Dried Powders

#### 3.1.1. Powder Yield

From an industrial point of view, the optimization of production yield represents a crucial factor in the selection of a drying agent, as it directly affects production costs and process efficiency. The effectiveness of a spray-drying process is considered satisfactory when the production yield is above 50% [19,24]. The spray-dried SHE (carrier-free) achieved 62.48% of powder yield, while significant changes were observed by adding different types of biopolymers (Table 1). Among the mono-carrier encapsulation systems, the samples prepared with CDs showed significantly higher drying yield compared to the conventional carriers (no statistically significant difference between BCD and HPBCD). The lowest yield was noticed with WP addition. In contrast, the macromolecule blends of WP/BCD and WP/HPBCD demonstrated the highest efficacy as carriers, exhibiting yields of 73.96% and 72.50%, respectively. Similarly, the admixtures of MD/BCD and MD/HPBCD led to significantly higher drying results (66.68% yields for BCD and 63.16% yields for HPBCD) compared to the drying yield of MD used as a single carrier (58.15%). These findings unequivocally indicate a synergistic effect between CDs and conventionally used biopolymers leading to higher drying efficiency.

The better yield achieved with CDs, compared to conventional carriers can be attributed to their ability to form inclusion complexes with the phytochemicals present in the extract. One of the main limitations of CDs as carriers is their relatively low loading capacity. Overcoming this limitation could be associated with the observed higher yields when CDs are combined with conventional biopolymers, especially WP. In particular, CDs, as molecules rich in -OH groups, can form hydrogen bonds with conventional carriers (especially with WP, while -NH_2_ and -COOH groups interact with the molecules), further improving the phyto-compounds uptake. Supramolecular and intermolecular complexes in dual-encapsulation systems can synergistically contribute to an improved integration of phyto-compounds with carriers, leading to a reduction in their loss during the drying process. It is well known that preventing the loss of low molecular weight molecules due to “stickiness and wall deposition phenomena” represents one of the most important requirements for efficient spray drying [31].

#### 3.1.2. Moisture Content

A powder quality parameter such as moisture content can be affected by various factors such as biopolymers, liquid feed, and inlet temperature during the drying process [27]. The verified moisture contents of the powdered extracts (ranging from 2.26 to 3.40%) were within the desired range to be considered as dried products. The observed low moisture contents (less than 5%) seem promising in terms of microbiological stability and extended shelf life [32,33,34]. In particular, the use of biopolymers led to the desired result of lower moisture content, as all encapsulated extracts, including those with mono- and dual-encapsulation systems, demonstrated lower moisture content compared to the pure extract (which was 3.43%). A statistically significant difference in moisture content was observed in the powder samples prepared with different carriers (Table 1). The extract macromolecule combination SHE+MD+BCD had the lowest moisture content (2.26%), while the powder prepared with WP had a higher value (3.40%). Similarly, the slightly higher moisture content of spray-dried vanillin with whey protein carrier was explained by Hundre et al., 2015 [35], through the formation of a surface crust that impedes the diffusion of water evaporation.

Mono-carrier encapsulation systems utilizing CDs exhibit significantly lower moisture content compared to conventional WP and MD systems. Blends of CDs with conventional carriers also demonstrated lower moisture compared to mono-carrier systems. A consistent trend in moisture content was observed for both WP- and MD-containing encapsulation systems: conventional mono-carriers (WP or MD) > blends of conventional carriers, and HPBCD > blends of conventional carriers and BCD. A similar pattern was observed in the spray drying of anthocyanin-rich blue maize (corn) waste, where microencapsulates with HPBCD as a mono-carrier, as well as a blend of MD and HPBCD, exhibited lower moisture compared to the conventional MD carrier [10]. In line with this, when vanillin was spray-dried, both with the BCD mono-carrier and with a blend of WP/BCD, it achieved a lower moisture content compared to the conventional WP carrier [32]. However, Pasrija et al., 2015 reported the highest moisture when BCD was used as a carrier for green tea extract encapsulation [36]. Similarly, Escobar-Avello et al., 2021 achieved a higher moisture content with the MD+HPBCD combinations for the microencapsulation of grape cane extract compared to pure extract [23].

#### 3.1.3. Bulk and Tapped Density, Carr Index, and Hausner Ratio

Bulk and tapped densities, flowability, and cohesiveness as powder quality parameters were determined and the results are presented in Table 1. These parameters may directly affect the properties of the final food or pharmaceutical product. The bulk density-BD values (Table 1) ranged from 0.20 g/mL (for SHE) to 0.27 g/mL (SHE+WP+HPBCD). All powders obtained with single-use carriers (MD, WP, BCD, HPBCD), as well as pure extract, showed statistically significantly lower values of bulk density compared to the carrier combinations. As expected, as well as previously reported by M. Jovanović, Ćujić-Nikolić, et al., 2021 [11], the increase in carrier concentration (here carrier combinations) is followed by an increase in bulk densities. The tapped densities (TD) (Table 1) ranged from 0.25 g/mL (for SHE) to 0.42 g/mL (SHE+MD+HPBCD). Among the mono-component carriers, BCD caused the highest TD. However, it was found that the moisture content did not show a direct effect on the bulk and tapped densities of the powdered extracts. Compared to our result for MD, slightly higher values for TD were reported by Caliskan & Dirim 2016 [25], for MD as a carrier (0.46–0.53 mg/mL). Dry powder flow classification (Carr index, CI) and cohesiveness parameters (Hausner ratio, HR) were estimated according to the previously reported study by Caliskan & Dirim 2016 [25]. The Carr index (CI) as the flowability measure, ranged from 20.7 to 41.17 for the examined micro-sized powders, and the pure spray-dried extract, and microencapsulates of SHE with the conventional biopolymers, MD and WP, were ranked as the powders with the best flowability. Slightly higher CI values were noticed for powders obtained using a single BCD and a combination of MD and HPBCD. The coherence of powders predicted on the Hausner ratio was analogous to intermediate cohesive powders (less than 1.4) for pure SHE, as well as SHE incorporated into MD or WP. Similar to CI results, the highest value of HR was observed for the SHE+BCD powder. The obtained values for bulk and tapped densities could be regarded as uniform microparticles produced by the spray-drying microencapsulation method which may be available for the production of final pharmaceutical products such as powders and tablets, as well as for functional food.

#### 3.1.4. Rehydration and pH

The time required for the complete dissolution of a powdered extract in a suitable solvent is commonly referred to as the rehydration time, whereas accurately determined time is necessary and has practical importance in the formulation process. Water serves as a primary dissolution medium for various oral products, including pharmaceuticals and foods.

The rehydration time of the studied samples was in the range of 48.51 s (SHE+BCD) to 128.71 s (SHE+MD), presented in Table 1. The rehydration time of the spray-dried extract without a carrier was measured to be 84.26 s. The inclusion of carriers had a statistically significant impact on the rehydration time, revealing discernible patterns of the carrier type’s influence on the powder rehydration. Among the mono-carrier encapsulates, powders formulated with conventional WP and MD carriers demonstrated prolonged rehydration times of 124.64 s and 128.17 s, respectively. Conversely, the utilization of HPBCD and BCD carriers resulted in a significant reduction of rehydration time, measuring 48.51 s and 61.17 s, respectively. In the case of powdered extracts with carrier blends, rehydration time values were significantly lower compared to those with conventional mono-carriers, which is in line with previous reports [10]. Particularly, the blends containing HPBCD exhibited shorter rehydration times compared to those containing BCD.

The presented results agree with the parameters of particle uniformity (Table 2) based on the laser diffraction method. Powders spray-dried with CDs and two carrier blends demonstrated a uniformity of approximately 0.5 (Table 2), revealing a homogenous mixture with lower energy needed for rehydration, since values closer to zero mean narrower distributions of the microparticles and better particle uniformity [37]. The higher uniformity values and longer rehydration time were observed for pure SHE, and SHE incorporated with MD and WP.

The acquired results of rehydration were similar to the previously reported data in the literature [21,25,38]. The finding that adding CDs shortens the rehydration time of powdered extracts could have practical implications in the formulations, such as instant products where rapid dissolution is desirable. In this context, it is worth noting that CDs as oligosaccharides with a low glycemic index can also be used as safe sweeteners, which makes them potential multifunctional excipients [16].

The pH values of the dissolved microencapsulated extracts in water ranged from 4.81 to 5.05, showing statistically significant differences among the samples depending on the carriers used. The addition of the carriers increased the pH value of pure SHE. Specifically, the samples containing SHE encapsulated with BCD, and MD+HPBCD displayed the highest pH values, which can be attributed to the presence of carrier combinations, increasing the concentration of carriers used, and their dilution effect compared with pure SHE.

All obtained pH values of reconstituted powders indicate that the microencapsulated extracts are within the suitable range for oral consumption.

#### 3.1.5. Hygroscopicity

Hygroscopicity represents the capacity of a material to absorb moisture from a high relative humidity environment. In pharmaceutical or food applications, it is linked to the powder porosity or the presence of amorphous sugars. Minimizing hygroscopicity is desirable in powder production to prevent excessive water absorption and stickiness. A powder with less than 20% hygroscopicity can be considered as a powder with low hygroscopicity [39].

**Figure 1:**
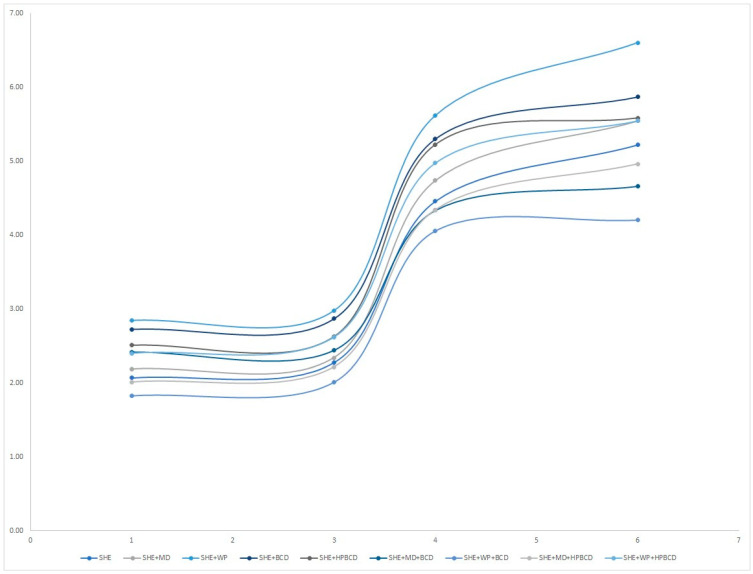
Hygroscopicity of *H. plicatum* spray-dried extract (SHE), and SHE microencapsulates with different carriers (maltodextrin (MD), whey protein (WP), *β*-cyclodextrin (BCD); hydroxypropyl-*β*-cyclodextrin (HPBCD)).

The water absorption of the SHEs was monitored after storage for 6 days, and results are presented in Figure 1 and Table S1. A difference in hygroscopicity values was recorded depending on the type of carrier used, but in all samples, was significantly lower than the mentioned limit of 20%. Based on these results, the obtained powder samples can be considered non-sticky and stable during the storage period. Among used biopolymers, the blend of macromolecule carriers, SHE+WP+BCD, exhibited the lowest hygroscopicity throughout the observed period, whereas the mono-carrier samples SHE+WP and SHE+BCD recorded the highest hygroscopicity values, emphasizing that blends of carriers can exhibit quite different features in comparison with the individual carriers.

Mono-carrier encapsulates exhibited higher hygroscopicity values, confirming that increasing the carrier concentration, when used in combinations, led to a decrease in hygroscopicity. This is in line with the results reported by Vidović et al., 2014 [24], who observed a decrease in the hygroscopicity of *Satureja montana* dry powder extract with an increasing percentage of MD as a carrier. This reduction in hygroscopicity can be attributed to the elevation of the glass transition temperature, as an increase in MD content leads to a higher glass transition temperature of the powder [24].

On the last monitoring day, the hygroscopicity did not change meaningfully, probably due to the saturation capacity of the powders.

### 3.2. Physicochemical Characterization of Spray-Dried Powders

#### 3.2.1. Particle Size Distribution

**Figure 2:**
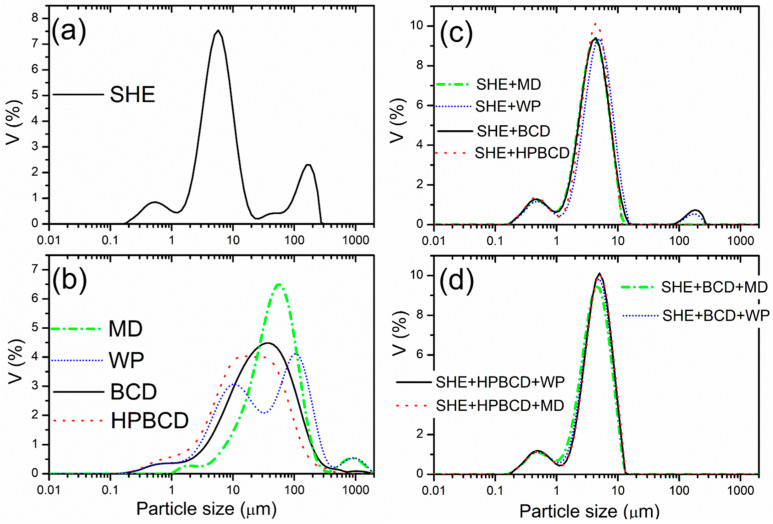
Particle size distribution of (**a**) *H. plicatum* spray-dried extract (SHE), (**b**) carriers of SHE microencapsulates, (**c**) SHE microencapsulates with mono-component carrier systems; and (**d**) SHE microencapsulates with dual-carrier systems.

The characterization of particles produced by the spray-drying technique with different biopolymers was determined by the laser diffraction method and presented in Table 2, as well as particle size distribution in Figure 2. Two (SHE microencapsulated in carrier combinations) or three distinct peaks (pure SHE and SHE microencapsulated in mono-carriers) represented the predominant particle size (Figure 2). The measured diameter of produced particles with biopolymers varied from 0.81 (d_10_) for SHE+HPBCD to 9.77 µm (d_90_) for SHE+MD, respectively, with the average diameter (d_50_) from 3.99 (SHE+MD) to 4.86 µm (SHE+MD+HPBCD), respectively (Table 2), classifying all obtained powders into the scale size of microparticles [6]. The SHE microparticles exhibited the highest particle diameter ranging from 2.08 (d_10_) to 136.71 µm (d_90_), while the mean average diameter was 6.49 μm. Spray drying of *H. plicatum* extract promoted the formation of small particles, and a biopolymer addition decreased the particle size in comparison with pure SHE. Pasrija et al., 2014 reported a decrease in particle size using a blend of MD+CD for green tea extract microencapsulation [36], while Escobar-Avello et al., 2021 reported an increase in grape cane extract mean particle diameter encapsulated with MD and HPBCD [23].

The mean diameter over the volume distribution D [4,3] ranged from 2.67 μm (SHE+MD+BCD) to 10.94 µm (SHE+HPBCD), and was 30.61 μm for the pure spray-dried extract. The values of the mean diameter of the spray-dried complexes of blueberry juice with 15% MD and 15% HPBCD were similar to those found in our study [21].

The mean surface area parameter, D [3,2], demonstrated the spheric surface area of microparticles, ranging from 2.11 to 2.44 for particles microencapsulated with carriers, and 3.29 for SHE, respectively. Low D [3,2] values for microencapsulated forms defined the spheric surface, especially with singly used MD and HPBCD.

The particle size distribution, characterized by a narrow variation in span values and low uniformity values, indicates uniform particles [40]. Dried pure *Helichrysum* extract exhibited the lowest particle size uniformity, confirmed with high span and particle uniformity values (Table 2). There were no observed significant differences in the PDI (span) values among microencapsulates. Notably, all samples containing CDs, whether mono-carriers or carrier blends, significantly improved the particle size uniformity. This observation was also previously confirmed by bulk and tapped density values and could be used for the future improvement of particle packing. On the other hand, samples with the highest particle size variation (SHE, SHE+MD, and SHE+WP with the highest uniformity values among microencapsulates) recorded low bulk and tapped density values; meanwhile, their flowability and cohesiveness were most appropriate (the lowest Hausner ratio and Carr’s index values).

#### 3.2.2. Fourier-Transform Infrared Analysis

**Figure 3:**
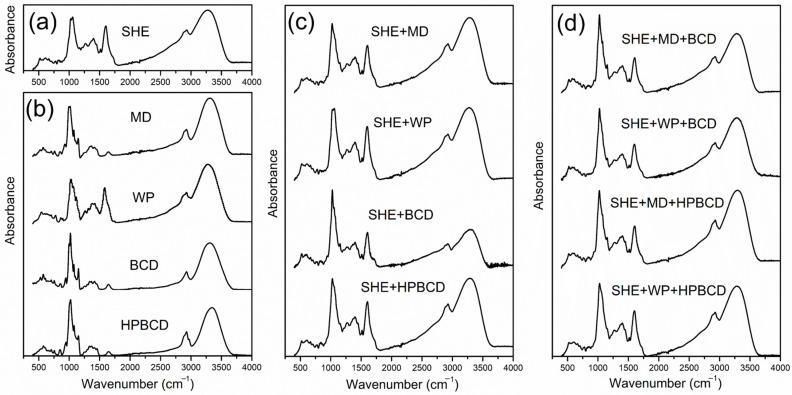
Fourier-transform infrared spectra of (**a**) *H. plicatum* spray-dried extract (SHE); (**b**) free carriers; and SHE microencapsulates with (**c**) mono-component carrier systems and (**d**) dual-carrier systems.

The formation of spray-dried complexes for each encapsulated sample was analyzed by the FTIR method. The FTIR spectra of the pure extract, biopolymers, and microencapsulated samples are shown in Figure 3. Infrared spectra of spray-dried samples possessed various meaningful peaks, originating from the *H. plicatum* extract and examined carriers (Figure 3).

The relevant bands with noticeable intensity around 1000 cm^−1^ could be related to the C-O stretching vibrations, while peaks between 1200 and 1500 cm^−1^ represented the contribution of the C-H bending vibrations [20]. The spectral peak around 1600 cm^−1^ corresponded to the characteristic C=O stretching vibration, confirming the presence of a carbonyl functional group [41]. This peak was visible in all SHE spray-dried powders, associated with polyphenol compounds present in the *H. plicatum* extract, as well as in the spectrum of the WP biopolymer, attributed to the peptide bond [41,42]. High-intensity peaks also appeared around 2800 cm^−1^ and 3250 cm^−1^_,_ associated with the C-H and -OH stretching vibrations, respectively [23]. As a result of the interaction with the biopolymers, the FTIR of SHE microencapsulates is slightly changed in the carbonyl and hydroxyl regions. It is noticeable that microencapsulates containing BCD possessed a slightly lower intensity of these peaks, suggesting the potential formation of hydrogen bond interactions. On the other hand, slightly higher intensity values of these peaks were observed for HPBCD microencapsulates, which could be attributed to the increase in the number of the functional groups associated with the C=O and –OH bonds in phenolic compounds.

Overall, it could be concluded that changes in the FTIR spectra demonstrated the presence of possible interactions among polymers and bioactive ingredients of *H. plicatum*, which could occur on the surface hydrophobic domain as well as through the formation of hydrogen bond interactions.

#### 3.2.3. Spray-Dried Powders Morphology

**Figure 4:**
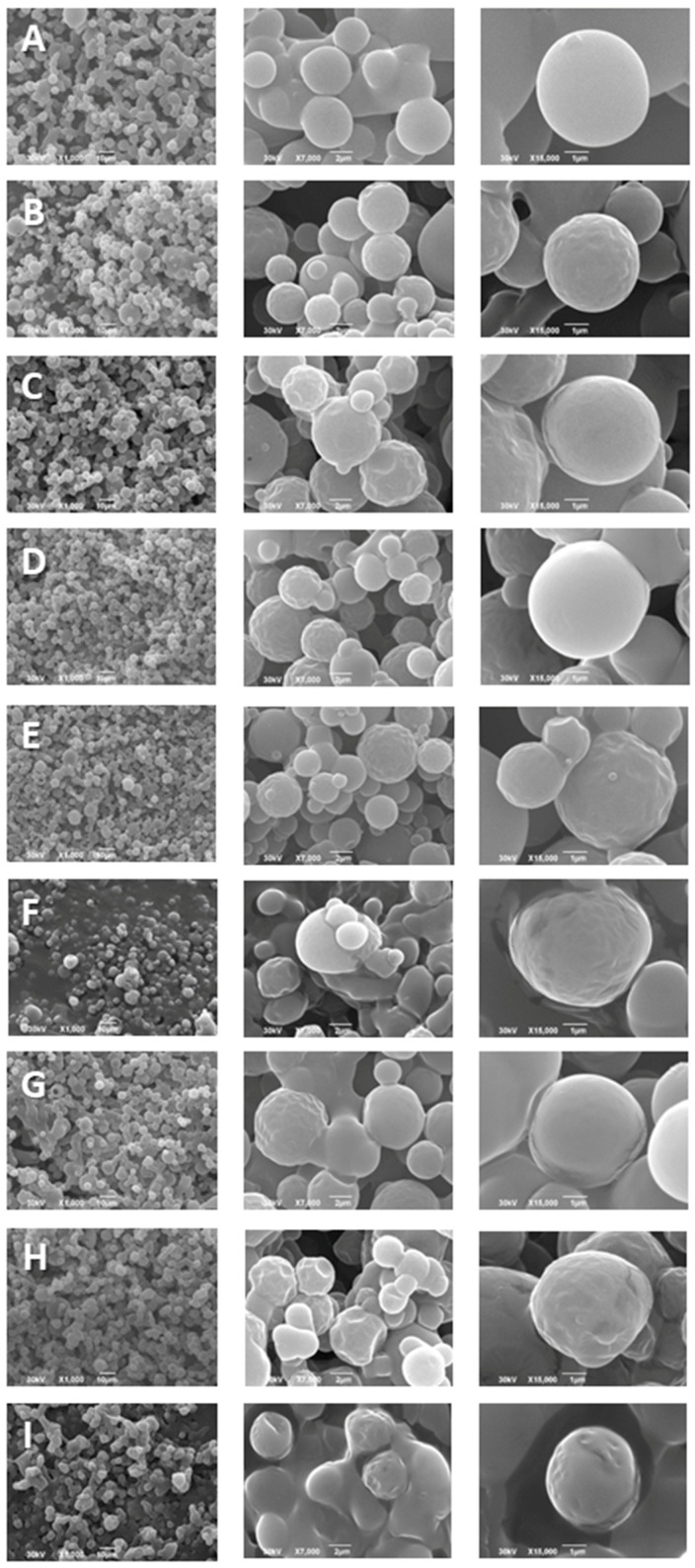
SEM micrographs of spray-dried *H. plicatum* powders at three different magnifications (each sample in 1000, 7000, and 15,000). (**A**) *H. plicatum* spray-dried extract (SHE) and microencapsulates of (**B**) SHE+MD; (**C**) SHE+WP; (**D**) SHE+HPCD; (**E**) SHE+BCD; (**F**) SHE+MD+BCD; (**G**) SHE+MD+HPCD; (**H**) SHE+WP+BCD; and (**I**) SHE+WP+HPCD.

The spray-dried microparticles morphology has been analyzed by SEM technique, as presented in Figure 4. Alterations in powder morphology produced with different biopolymers have been noticed. The spray-dried *H. plicatum* extract micrograph demonstrated powder with visible aggregates, while carrier–extract complexes were different from the original morphology of pure SHE. Biopolymer addition generally caused the production of clearly defined and separated particles. Microparticles produced with MD possessed tinny aggregations, but were less stretched and clearly defined in comparison to SHE, while microencapsulated SHE in WP retained slightly shrunken particles. Similar observations were noticed with spray-dried chokeberry extracts [20]. Microencapsulates of SHE+BCD were presented as an enlargement of small particles, spherical and with smooth surfaces, without observable destructions.

SHE+HPBCD microparticles have been noticeably separated and are smaller in comparison with the particles produced with BCD, following laser diffraction analysis (Table 2). Microparticles produced with CDs demonstrated association in cluster units of small particles, probably due to the CD tendency to agglomerate in polar mediums such as extracts [43]. This observation is even more evident when SHE is microencapsulated in the macromolecule combination WP+HPBCD, with a greater advantage in terms of powder morphology, influenced by the shapes of microencapsulates. In general, the macromolecule biopolymer combinations exhibited the production of more spherical particles, which were less aggregated compared to a mono-carrier application [38], which showed more irregular morphology in encapsulated carvacrol using HPBCD, established by freeze drying and kneading. There was no observed crystalline structure in the obtained SHE microencapsulates, and no visible destruction, implicating the successful preservation of *H. plicatum* extract.

#### 3.2.4. Thermal Characteristics of Micro-Powders

**Figure 5:**
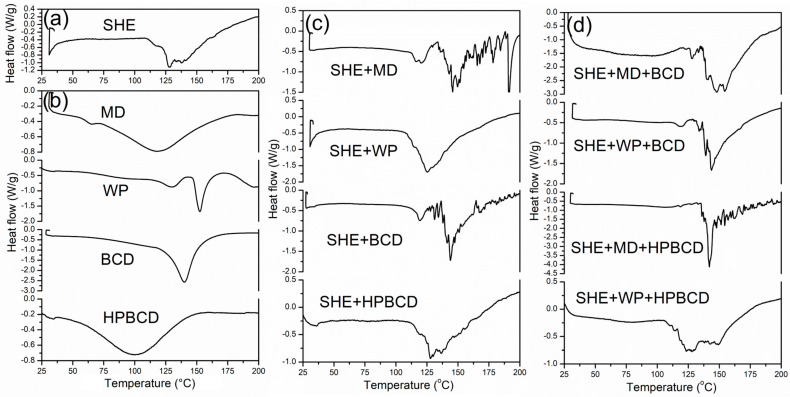
DSC thermal diagrams of (**a**) *H. plicatum* spray-dried extract (SHE); (**b**) free carriers; and SHE microencapsulates with (**c**) mono-component carrier systems and (**d**) dual-carrier systems.

For the thermal stability determination of spray-dried *H. plicatum* extract and the microencapsulates produced using four different carriers, the DSC analysis has been provided. Figure 5 demonstrates the DSC thermograms for SHE and SHE microencapsulates. Since DSC analysis could supervise the changes in thermal characteristics of the microencapsulation process, peak transition temperatures and the enthalpy changes are presented in Table 3. DSC curves (Figure 5) were predominantly observed to have an initial endotherm peak, mostly correlated with the evaporation of water, and volatile compounds characterized with low enthalpy due to the powder’s low moisture content [6,44]. Characteristic and noticeable endothermic peak maximums were present among 125 and 150 °C for the examined spray-dried samples, possibly originating from polyphenols transitioning and carriers liquifying. The thermal evaporation of the microencapsulated SHE in biopolymers was repositioned to the higher temperatures, associated with higher enthalpy changes probably due to the host–guest interactions of the biopolymer and polyphenolic compounds. The MD and BCD addition increased degradation temperatures compared to pure SHE, although these microencapsulates demonstrated several temperature changes compared to clearly defined endothermic peaks among other microencapsulates. The SHE microencapsulated in MD/BCD biopolymer blends signified the highest temperature degradation as well as the highest energy change. The addition of the high molecular weight biopolymers to the feed solution for the microencapsulation process resulted in the degradation temperature elevation of the microencapsulates, compared to their unencapsulated form. According to the literature, carbohydrates (MD and CDs), can preserve the active compound’s structure [45,46], due to their chemical composition and high numbers of hydroxyl and carbonyl groups. These findings follow the results of spray-dried wild thyme extract [47]. There were no observed exothermic peaks, associated with the amorphization process of extract and biopolymers, but endothermic peaks enlarged at higher temperatures [6]. Concerning the results of thermal analysis, the examined micro-powders could be respected as systems with good thermal stability (around 200 °C), a temperature area important for food and pharmaceutical processing.

### 3.3. Chemical Characterization of Microparticles

#### 3.3.1. Analysis of Total Bioactives Content and Encapsulation Efficiency

Encapsulation efficiency represents a crucial parameter for the success of the microencapsulation process, demonstrating the real quantity or actual load of encapsulated bioactives. The microencapsulation process performed by spray drying resulted in powders with high encapsulation efficiency, with more than 80% of *H. plicatum* extract polyphenols microencapsulated in biopolymers (Table 4). The spray-dried SHE was not microencapsulated with carriers and cannot be related to encapsulation efficiency, but rather to drying efficiency (97.325%), not influenced by carrier addition. The obtained microencapsulates reached encapsulation efficiencies ranging from 80.07% (SHE+MD+HPBCD) to 96.45% (SHE+HPBCD). Among all observed biopolymers, HPBCD showed the best characteristics for entrapping the *H. plicatum* polyphenols, from the standpoint of successful encapsulation. Despite the dilution effect, SHE+HPBCD exhibited a similar EE to that of the pure SHE. Moreover, alongside HPBCD as a novel biopolymer, BCD also showed a high EE (92.36%) compared to the conventional ones. According to the literature, cyclodextrins and their complexes can form water-soluble aggregates in aqueous extracts, and these aggregates can entrap bioactive compounds by non-inclusion complexation or micelle-like structures [48]. Therefore, it could be concluded that specific cavities and molecular shapes distinguish CDs as superior candidates for phenolic compound preservation. Additionally, polar side-chains such as the additional hydroxypropyl (HP) groups of HPBCD may enhance the encapsulation of polar compounds such as polyphenols compared to the BCD [49]. An analogous observation was reported by Şahin-Nadeem et al. for sage polyphenols microencapsulated in MD, gum arabic, and β-CD [50]. They showed the highest EE when the carrier was added at the lowest concentration.

One of the tremendous advantages of the microencapsulation process in foods and pharmaceuticals is the high concentration of bioactive compounds they can incorporate. The content of total polyphenols in LHE calculated on dry weight was 0.804 mg GAE/g, while this content per gram of microencapsulates was even 125-fold higher, related to SHE+HPBCD. This unique property generates microencapsulates as effective preparations for bioactives preservation. All of the examined carriers provided good entrapment efficiency, confirming spray drying as a suitable preservation technique for *H. plicatum* extract. The SHE powder had the highest polyphenol content (106.32 mg GAE/g), while microencapsulates were in the range from 83.25 (SHE+WP+HPBCD) to 100.28 (SHE+HPBCD) mg GAE/g (Table 4). In general, all obtained powders achieved high TPC, primarily with the highest values observed for microencapsulates produced with CDs. Biopolymer blends were associated with the dilution of bioactives in spray-dried powders, in agreement with EE. Similar results were demonstrated in our previously published study on microencapsulated willow gentian extract with different carriers [11], followed by the other authors for the microencapsulation of willow bark, sage, and yarrow [24,50,51].

A similar pattern to TPC was observed for the content of total flavonoids. The highest TFC was found in the dried SHE, followed by the SHE microencapsulated in HPBCD, without a statistically significant difference, despite dilution effect (Table 4). Outstanding mono-component carriers achieved similar values of encapsulated TFC. However, a higher concentration of carrier combinations led to a decrease in TFC, due to the dilution effect.

Overall, the obtained results revealed that all microencapsulated powders reached high levels of total bioactive principles, suggesting good preservation by the studied polymers.

#### 3.3.2. HPLC Analysis of Individual Compounds in Microencapsulates

*H. plicatum* is a well-known source of potent phenolic compounds, with flavonoids being the most predominant secondary metabolites. The spray-dried microparticles with and without carriers were characterized by the HPLC method, and nine individual phenolics, including isoquercitrin, kaempferol, kaempferol-3-*O*-glucoside, apigenin, apigenin-7-*O*-glucoside, and naringenin and its three glycosylated derivatives were detected, which is consistent with previous findings in the literature [2,3,49] (Table 5, Figure S1). In the prepared powders, kaempferol-3-*O*-glucoside was the most prominent compound among all others, with the content ranging from 16.29 to 22.59 mg/g. The highest value was observed in the carrier-free powder, while slightly lower values were detected in the mono- and dual-component encapsulation systems, respectively. This finding is consistent with the previously described dilution effect due to an increase in the carrier amount. However, it is noteworthy that microencapsulated systems with HPBCD addition showed the greatest capability to preserve bioactive compounds from *H. plicatum* extract. Among the mono-component carrier systems, SHE+HPBCD possesses the most promising polyphenolic profile. Even compared to pure SHE, it was characterized by the highest content of detected aglycones (kaempferol, apigenin, and naringenin), while slightly lower values were observed for their glycosylated forms despite the dilution effect (Table 5). Considering results obtained for dual-encapsulation systems with BCD, there was no statistically significant difference in the content of glycosylated forms, while SHE+MD+BCD possessed a significantly higher content of aglycones (Table 5). In contrast, an analysis of the dual-encapsulation systems with HPBCD revealed that the mixture of WP and HPBCD had the highest potential to preserve bioactive compounds from *H. plicatum* extract. Overall, as shown in Table 5, it can be concluded that both SHE+MD+BCD and SHE+WP+HPBCD have similar efficacy, without any statistically significant difference in the content of individual compounds. Taking into account various experimental factors, including solvent type and concentrations, the extraction method, and the carriers used, the extraction yields of bioactive compounds can vary considerably when analyzing different plant materials. In previous studies, Bigović et al. have examined ethyl acetate-ethanol H. plicatum flower extracts, and high amounts of naringenin, apigenin, and kaempferol were detected after the deglycosylation process [30]. Furthermore, kaempferol-3-*O*-glucoside was also detected in the methanolic extracts of H. italicum flowers, in a concentration of 2.61 mg/100 g DW [52]. The flavanols apigenin, apigenin-7-glucoside, and naringenin have also been isolated from various Helichrysum species and have been shown to possess potent antioxidant capacities [53].

The obtained findings are consistent with the aforementioned analysis of TPC, TFC, and EE and confirm the high efficacy of CD-based encapsulated systems for the successful preservation of bioactive compounds.

#### 3.3.3. PCA Analysis

**Figure 6:**
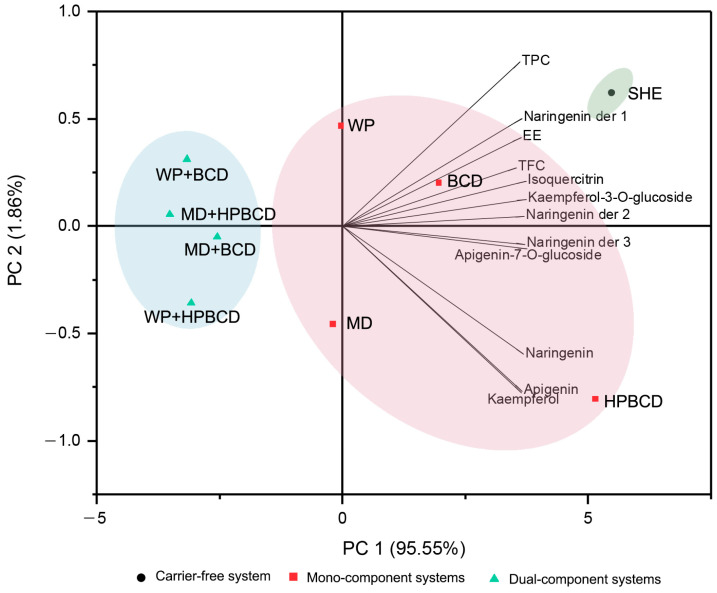
The principal component analysis (PCA) performed with two factors explaining 97.41% of the total variance.

To generally evaluate the behavior of different wall materials based on the results of encapsulation efficiency and the content of total and individual phenolic and flavonoid compounds, a PCA analysis was performed. The results presented in Figure 6 revealed that the variables could be reduced to two components, explaining 97.41% of the total variance. The first component (PC1) represents 95.55% of the variability, while the second component (PC2) represents 1.86%. The obtained findings indicate the great superiority of HPBCD compared to the other examined wall materials, which is reflected in a high EE and a high content of total and individual bioactive compounds, especially aglycones. This could be the result of the effective encapsulation properties of the HP-β-CD cavity, as well as the presence of the HP groups, which could enhance the formation of the hydrogen bond interactions and the overall stability of the inclusion complexes. Not only the lower polarity but also the specific geometrical properties of the aglycones could potentiate their higher affinity for incorporation into the non-polar cavity of HP-β-CD compared to their glycosylated forms [13]. Furthermore, PCA analysis for the mono-encapsulation systems found that protein biopolymer, WP, and BCD had a higher impact on TPC, TFC, and the content of individual glycosides compared to the carbohydrate, MD polymer. The same trend was also observed in the dual microencapsulates with BCD, which showed a greater affinity of WP+BCD for the glycoside and MD+BCD for the aglycone components. However, this was not the case for HPBCD mixtures, indicating higher compatibility between WP and HPBCD to potentate the preservation of both glycosides and aglycones.

## 4. Conclusions

The flowers of *Helichrysum plicatum* DC., with evident spasmolytic, antioxidant, and antimicrobial activity, have a long tradition in the treatment of various ailments associated with gastrointestinal disorders. The practicability of the spray-drying process of *Helichrysum plicatum* flower extract reflects the enhancement of functional powder properties. The pure spray-dried *Helichrysum* extract achieved a high concentration of bioactive compounds; however, its technological properties (bulk and tapped density, hygroscopicity, and particle size), and powder morphology, indicate a less homogenous powder. The addition of conventional (MD, WP) and innovative biopolymers (BCD, HPBCD) and their macromolecule combinations resulted in powders with improved technological performance and a high preservation efficiency of the bioactive compounds. The HPBCD addition remarkably improved all powders’ properties, technological and physicochemical. Analysis of particle morphology revealed that the addition of biopolymer-macromolecule mixtures resulted in the production of more spherical and less aggregated particles compared to the mono-carrier systems. DSC analysis indicated the great potential of the carrier blends used to protect the spray-dried particles against temperature degradation. The microencapsulation process performed resulted in micro-sized powders with a high encapsulation efficiency of polyphenolic compounds of up to 80%. Phytochemical analysis revealed that the total phenolic and flavonoid content in the spray-dried powders varied from 83.25 to 106.32 mg GAE/g, and 15.66 to 21.85 mg catechin/g, respectively, with kempferol-3-*O*-glucoside being the most prominent compound. Among all combinations, microencapsulated systems with HPBCD showed the greatest ability to preserve bioactive compounds from *H. plicatum* extract, reflected in the high encapsulation efficiency, and in the content of total and individual phenolic compounds. Both the proposed mono- and dual-encapsulation systems of *Helichrysum plicatum* L. flower extract offer numerous opportunities for further applications in food or pharmaceuticals.

## Figures and Tables

**Table 1:** Technological parameters of spray-dried *Helichrysum* extract (SHE) and SHE microencapsulates.

Samples	Yield	Moisture	BD	TD	CI	HR	Rehydration	pH
	(%)	(%)	(g/mL)	(g/mL)			(s)	
SHE	62.48 ± 1.82 bc	3.43 ± 0.20 a	0.20 ± 0.01 d	0.25 ± 0.01 d	20.92 ± 0.51 e	1.26 ± 0.01 e	84.26 ± 1.74 c	4.68 ± 0.01 e
SHE+MD	58.15 ± 1.69 cd	2.94 ± 0.02 c	0.22 ± 0.02 cd	0.27 ± 0.02 d	20.70 ± 0.46 e	1.26 ± 0.01 e	128.70 ± 1.24 a	4.81 ± 0.00 d
SHE+WP	55.89 ± 1.63 d	3.40 ± 0.01 ab	0.22 ± 0.01 cd	0.27 ± 0.01 d	21.29 ± 0.45 e	1.27 ± 0.01 e	124.64 ± 8.06 a	4.83 ± 0.01 d
SHE+BCD	60.24 ± 1.76 cd	2.85 ± 0.13 cd	0.23 ± 0.00 bc	0.40 ± 0.00 ab	41.17 ± 0.69 a	1.70 ± 0.02 a	61.17 ± 2.47 d	5.05 ± 0.01 a
SHE+HPBCD	60.81 ± 1.77 cd	2.52 ± 0.02 de	0.23 ± 0.00 bc	0.35 ± 0.00 c	33.95 ± 0.20 c	1.51 ± 0.00 c	48.51 ± 1.12 e	4.86 ± 0.01 c
SHE+MD+BCD	66.68 ± 1.94 b	2.26 ± 0.14 e	0.26 ± 0.01 ab	0.37 ± 0.02 bc	30.85 ± 0.85 d	1.45 ± 0.02 d	102.27 ± 3.01 b	4.82 ± 0.02 d
SHE+WP+BCD	73.96 ± 2.16 a	2.71 ± 0.21 cd	0.25 ± 0.00 ab	0.40 ± 0.00 ab	36.62 ± 0.73 b	1.58 ± 0.02 b	106.70 ± 6.20 b	4.83 ± 0.01 d
SHE+MD+HPBCD	63.16 ± 1.84 bc	2.89 ± 0.16 cd	0.25 ± 0.01 ab	0.42 ± 0.03 a	40.28 ± 0.75 a	1.67 ± 0.02 a	65.18 ± 3.32 d	5.02 ± 0.01 b
SHE+WP+HPBCD	72.50 ± 2.12 a	3.02 ± 0.10 bc	0.27 ± 0.01 a	0.40 ± 0.00 ab	34.17 ± 0.83 c	1.52 ± 0.02 c	79.01 ± 1.11 c	4.86 ± 0.00 c

Means followed by different letters are significantly different according to the post hoc Duncan’s test al a level of *p* ≤ 0.05. SHE—spray-dried *Helichrysum* extract, MD—maltodextrin, WP—whey protein, BCD—β-cyclodextrin, HPBCD—hydroxypropyl-β-cyclodextrin, BD—bulk density, TD—tapped density, CI—Carr index, HR—Hausner ratio.

**Table 2:** Particle size of spray-dried *Helichrysum* extract (SHE) and microencapsulates.

Samples	d_10_^a^	d_50_^b^	d_90_	Span ^c^	D [4,3]	D [3,2]	Uniformity
SHE	2.08 ± 0.29 de	6.49 ± 0.86 d	136.71 ± 17.28 b	20.74 ± 2.08 a	30.61 ± 3.15 c	3.29 ± 0.41 cd	4.15 ± 0.22 a
SHE+MD	1.04 ± 0.15 ef	3.99 ± 0.27 d	7.78 ± 0.58 d	1.69 ± 0.15 d	4.30 ± 0.57 d	2.11 ± 0.22 d	2.03 ± 0.24 bc
SHE+WP	1.31 ± 0.07 ef	4.81 ± 0.60 d	9.77 ± 0.77 d	1.76 ± 0.15 d	9.77 ± 1.27 d	2.42 ± 0.14 d	1.76 ± 0.26 cd
SHE+BCD	1.09 ± 0.17 ef	4.19 ± 0.51 d	8.96 ± 0.93 d	1.88 ± 0.22 d	10.94 ± 1.37 d	2.20 ± 0.14 d	0.49 ± 0.04 e
SHE+HPBCD	0.81 ± 0.10 f	4.23 ± 0.48 d	7.82 ± 0.64 d	1.63 ± 0.13 d	4.45 ± 0.29 d	2.12 ± 0.32 d	0.46 ± 0.06 e
SHE+MD+BCD	1.24 ± 0.14 ef	4.35 ± 0.47 d	8.39 ± 1.28 d	1.65 ± 0.16 d	2.67 ± 0.38 d	2.31 ± 0.17 d	0.49 ± 0.04 e
SHE+WP+BCD	1.28 ± 0.17 ef	4.62 ± 0.26 d	8.70 ± 0.45 d	1.61 ± 0.13 d	4.91 ± 0.35 d	2.38 ± 0.34 d	0.47 ± 0.04 e
SHE+MD+HPBCD	1.30 ± 0.08 ef	4.86 ± 0.64 d	9.04 ± 1.23 d	1.59 ± 0.10 d	5.13 ± 0.45 d	2.44 ± 0.12 d	0.46 ± 0.06 e
SHE+WP+HPBCD	1.22 ± 0.13 ef	4.77 ± 0.45 d	8.86 ± 1.25 d	1.60 ± 0.16 d	5.04 ± 0.48 d	2.39 ± 0.16 d	0.46 ± 0.03 e

^a^ d_10_, d_50_, and d_90_ represent the sizes where 10%, 50%, and 90% of the particles are smaller than the remaining particles; ^b^ mean diameter; ^c^ calculated as (d_90_ − d_10_)/d_50_; Means followed by different letters are significantly different according to the post hoc Duncan’s test at a level of *p* ≤ 0.05. SHE—spray-dried *Helichrysum* extract, MD—maltodextrin, WP—whey protein, BCD—β-cyclodextrin, HPBCD—hydroxypropyl-β-cyclodextrin.

**Table 3:** The transition temperatures (T) and enthalpy changes (∆H) of spray-dried SHE, SHE microencapsulates, and biopolymers.

Samples	T (°C)	∆H (J/g)
SHE	128.57 ± 17.73 b	186.28 ± 32.65 bc
SHE+MD	145.77 ± 25.63 ab	158.23 ± 13.30 bcd
SHE+WP	125.45 ± 9.69 b	262.68 ± 21.37 a
SHE+BCD	143.99 ± 24.74 ab	154.41 ± 8.83 bcd
SHE+HPBCD	127.87 ± 15.72 b	180.56 ± 14.34 bc
SHE+MD+BCD	154.42 ± 25.04 ab	155.14 ± 12.64 bcd
SHE+WP+BCD	143.77 ± 21.88 b	156.04 ± 24.10 bcd
SHE+MD+HPBCD	141.97 ± 17.96 b	269.96 ± 14.66 a
SHE+WP+HPBCD	127.88 ± 21.30 b	170.24 ± 21.66 bc
MD	118.60 ± 19.06 b	126.41 ± 21.44 cd
WP	200.76 ± 19.55 a	104.28 ± 5.65 d
BCD	140.12 ± 10.33 ab	198.51 ± 33.15 b
HPBCD	100.31 ± 10.89 b	177.93 ± 21.56 bc

Means followed by different letters are significantly different according to the post hoc Duncan’s test at a level of *p* ≤ 0.05. SHE—spray-dried *H. plicatum* extract, MD—maltodextrin, WP—whey protein, BCD—β-cyclodextrin, HPBCD—hydroxypropyl-β-cyclodextrin.

**Table 4:** Encapsulation efficiency (EE) and total compound contents of spray-dried *Helichrysum* extract (SHE) and SHE microencapsulates.

Samples	EE (%)	TPC (mg GAE/g) *	TFC (mg Catechin/g) **
SHE	97.25 ± 1.54 a ^#^	106.32 ± 3.60 a	21.85 ± 0.52 a
SHE+MD	84.56 ± 0.43 de	87.93 ± 0.44 de	19.00 ± 0.36 bc
SHE+WP	87.92 ± 2.04 cd	91.42 ± 2.13 cd	19.54 ± 0.36 c
SHE+BCD	92.36 ± 2.49 bc	96.03 ± 2.59 bc	19.34 ± 0.16 bc
SHE+HPBCD	96.45 ± 0.20 ab	100.28 ± 0.21 b	21.55 ± 0.03 a
SHE+MD+BCD	82.82 ± 0.83 e	85.92 ± 0.66 de	17.63 ± 0.02 cd
SHE+WP+BCD	82.40 ± 2.25 e	85.68 ± 2.33 e	16.30 ± 1.23 de
SHE+MD+HPBCD	80.07 ± 0.58 e	83.25 ± 0.60 e	16.08 ± 1.40 de
SHE+WP+HPBCD	81.55 ± 2.35 e	84.79 ± 2.44 e	15.66 ± 0.06 e

* mg gallic acid equivalents/g of microencapsulates; ** mg catechin equivalents/g of microencapsulates; ^#^ drying efficiency. Means followed by different letters are significantly different according to the post hoc Duncan’s test at a level of *p* ≤ 0.05. SHE—spray-dried *Helichrysum* extract, MD—maltodextrin, WP—whey protein, BCD—β-cyclodextrin, HPBCD—hydroxypropyl-β-cyclodextrin, TPC—total polyphenols content, TFC—total flavonoids content.

**Table 5:** The content of individual compounds of spray-dried *Helichrysum* extract (SHE) and SHE microencapsulates.

Samples	Isoquercitrin *	Kaempferol-3-*O*-glucoside	Kaempferol	Naringenin	Apigenin7-*O*- glucoside	Apigenin	Naringenin Derivate 1	Naringenin Derivate 2	Naringenin Derivate 3
SHE	2.23 ± 0.024 a	22.59 ± 0.270 a	0.44 ± 0.013 b	0.58 ± 0.020 b	3.79 ± 0.048 a	2.49 ± 0.036 b	7.83 ± 0.140 a	11.19 ± 0.267 a	19.62 ± 0.260 a
SHE+MD	1.80 ± 0.004 d	18.62 ± 0.067 d	0.26 ± 0.001 d	0.25 ± 0.002 d	3.12 ± 0.011 c	2.04 ± 0.009 d	6.30 ± 0.030 d	9.19 ± 0.027 c	16.13 ± 0.023 d
SHE+WP	1.82 ± 0.001 d	18.77 ± 0.002 d	0.18 ± 0.001 e	0.20 ± 0.009 e	3.13 ± 0.001 c	1.86 ± 0.002 e	6.32 ± 0.001 d	9.30 ± 0.009 c	16.58 ± 0.018 c
SHE+BCD	1.95 ± 0.004 c	19.88 ± 0.064 c	0.31 ± 0.003 c	0.34 ± 0.003 c	3.32 ± 0.010 b	2.16 ± 0.007 c	6.75 ± 0.026 c	10.28 ± 0.027 b	17.93 ± 0.022 b
SHE+HPBCD	2.12 ± 0.014 b	21.78 ± 0.094 b	0.51 ± 0.010 a	0.72 ± 0.018 a	3.76 ± 0.017 a	2.72 ± 0.018 a	7.04 ± 0.045 b	11.15 ± 0.068 a	19.97 ± 0.121 a
SHE+MD+BCD	1.59 ± 0.011 e	16.61 ± 0.061 e	0.13 ± 0.010 f	0.04 ± 0.011 f	2.76 ± 0.010 d	1.67 ± 0.019 f	5.44 ± 0.028 de	8.66 ± 0.028 d	15.46 ± 0.272 e
SHE+WP+BCD	1.57 ± 0.009 ef	16.47 ± 0.077 ef	0.04 ± 0.013 g	0.01 ± 0.000 g	2.72 ± 0.015 de	1.50 ± 0.013 g	5.42 ± 0.042 e	8.68 ± 0.072 d	15.18 ± 0.086 ef
SHE+MD+HPBCD	1.54 ± 0.005 f	16.29 ± 0.045 f	0.04 ± 0.015 g	0.01 ± 0.000 g	2.69 ± 0.006 e	1.46 ± 0.004 g	5.37 ± 0.012 e	8.70 ± 0.014 d	15.08 ± 0.040 ef
SHE+WP+HPBCD	1.56 ± 0.012 ef	16.44 ± 0.066 ef	0.14 ± 0.002 f	0.03 ± 0.001 fg	2.74 ± 0.006 de	1.69 ± 0.027 f	5.43 ± 0.018 e	8.51 ± 0.022 d	14.87 ± 0.067 ef

* mg/g of microbeads. Means followed by different letters are significantly different according to the post hoc Duncan’s test at a level of *p* ≤ 0.05. SHE—spray-dried *Helichrysum* extract, MD—maltodextrin, WP—whey protein, BCD—β-cyclodextrin, HPBCD—hydroxypropyl-β-cyclodextrin.

## Data Availability

The data is contained within the manuscript.

## Supplementary Materials

The following supporting information can be downloaded at: https://www.mdpi.com/article/10.3390/pharmaceutics16070861/s1, Table S1: Hygroscopicity of spray-dried *Helichrysum* extract (SHE) and microencapsulates; Figure S1: The high-performance liquid chromatography (HPLC) spectrum of the *H. plicatum* extract powder: 1,2,3-glycosylated naringenin derivatives, 4-isoquercitrin, 5-kaempferol-3-*O*-glucoside, 6-apigenin-7-*O*-glucoside, 7-naringenin, 8-apigenin, 9-kaempferol.

## References

[1] Akaberi M., Sahebkar A., Azizi N., Emami S.A. (2019). Everlasting Flowers: Phytochemistry and Pharmacology of the Genus Helichrysum. Ind. Crops Prod..

[2] Vujić B., Vidaković V., Jadranin M., Novaković I., Trifunović S., Tešević V., Mandić B. (2020). Composition, Antioxidant Potential, and Antimicrobial Activity of Helichrysum Plicatum DC. Various Extracts. Plants.

[3] Bigovic D., Stevic T., Jankovic T., Noveski N., Radanovic D., Pljevljakusic D., Djuric Z. (2017). Antimicrobial Activity of Helichrysum Plicatum DC. Hem. Ind..

[4] Bigovic D., Brankovic S., Kitic D., Radenkovic M., Jankovic T., Savikin K., Zivanovic S. (2010). Relaxant Effect of the Ethanol Extract of Helichrysum Plicatum (Asteraceae) on Isolated Rat Ileum Contractions. Molecules.

[5] Jovanović M., Drinić Z., Bigović D., Alimpić-Aradski A., Duletić-Laušević S., Šavikin K. (2020). In Vitro Antineurodegenerative Activity and in Silico Predictions of Blood-Brain Barrier Penetration of Helichrysum Plicatum Flower Extract. Lek. Sirovine.

[6] Castro-López C., Espinoza-González C., Ramos-González R., Boone-Villa V.D., Aguilar-González M.A., Martínez-Ávila G.C.G., Aguilar C.N., Ventura-Sobrevilla J.M. (2021). Spray-Drying Encapsulation of Microwave-Assisted Extracted Polyphenols from Moringa Oleifera: Influence of Tragacanth, Locust Bean, and Carboxymethyl-Cellulose Formulations. Food Res. Int..

[7] Samborska K., Boostani S., Geranpour M., Hosseini H., Dima C., Khoshnoudi-Nia S., Rostamabadi H., Falsafi S.R., Shaddel R., Akbari-Alavijeh S. (2021). Green Biopolymers from By-Products as Wall Materials for Spray Drying Microencapsulation of Phytochemicals. Trends Food Sci. Technol..

[8] Šavikin K., Nastić N., Janković T., Bigović D., Miličević B., Vidović S., Menković N., Vladić J. (2021). Effect of Type and Concentration of Carrier Material on the Encapsulation of Pomegranate Peel Using Spray Drying Method. Foods.

[9] Gharsallaoui A., Roudaut G., Chambin O., Voilley A., Saurel R. (2007). Applications of Spray-Drying in Microencapsulation of Food Ingredients: An Overview. Food Res. Int..

[10] Ćujić Nikolić N., Žilić S., Simić M., Nikolić V., Živković J., Marković S., Šavikin K. (2023). Microencapsulates of Blue Maize Polyphenolics as a Promising Ingredient in the Food and Pharmaceutical Industry: Characterization, Antioxidant Properties, and In Vitro-Simulated Digestion. Foods.

[11] Jovanović M., Ćujić-Nikolić N., Drinić Z., Janković T., Marković S., Petrović P., Šavikin K. (2021). Spray Drying of Gentiana Asclepiadea L. Root Extract: Successful Encapsulation into Powders with Preserved Stability of Bioactive Compounds. Ind. Crops Prod..

[12] Liu Z., Ye L., Xi J., Wang J., Feng Z. (2021). Cyclodextrin Polymers: Structure, Synthesis, and Use as Drug Carriers. Prog. Polym. Sci..

[13] Radan M., Živković J., Nedeljković S.K., Janković T., Lazarević Z., Bigović D., Šavikin K. (2023). Influence of Hydroxypropyl-β-Cyclodextrin Complexation on the Extraction Efficiency of Rutin, Quercetin and Total Polyphenols from Fagopyrum Esculentum Moench. Sustain. Chem. Pharm..

[14] Sharif N., Golmakani M.-T., Hajjari M.M., Aghaee E., Ghasemi J.B. (2021). Antibacterial Cuminaldehyde/Hydroxypropyl-β-Cyclodextrin Inclusion Complex Electrospun Fibers Mat: Fabrication and Characterization. Food Packag. Shelf Life.

[15] Agrawal R., Gupta V. (2012). Cyclodextrins—A Review on Pharmaceutical Application for Drug Delivery. Int. J. Pharm. Front. Res..

[16] Chew S.C., Tan C.P., Nyam K.L. (2018). Microencapsulation of Refined Kenaf (*Hibiscus Cannabinus* L.) Seed Oil by Spray Drying Using β-Cyclodextrin/Gum Arabic/Sodium Caseinate. J. Food Eng..

[17] Stella V.J., He Q. (2008). Cyclodextrins. Toxicol. Pathol..

[18] Gidwani B., Vyas A. (2015). A Comprehensive Review on Cyclodextrin-Based Carriers for Delivery of Chemotherapeutic Cytotoxic Anticancer Drugs. Biomed. Res. Int..

[19] Jovanović M., Drinić Z., Bigović D., Zdunić G., Mudrić J., Šavikin K. (2021). Effect of Carrier Type on the Spray-Dried Willowherb (*Epilobium Angustifolium* L.) Leaves Extract Powder Properties and Bioactive Compounds Encapsulation. Lek. Sirovine.

[20] Ćujić-Nikolić N., Stanisavljević N., Šavikin K., Kalušević A., Nedović V., Samardžić J., Janković T. (2019). Chokeberry Polyphenols Preservation Using Spray Drying: Effect of Encapsulation Using Maltodextrin and Skimmed Milk on Their Recovery Following in Vitro Digestion. J. Microencapsul..

[21] Wilkowska A., Ambroziak W., Czyżowska A., Adamiec J. (2016). Effect of Microencapsulation by Spray Drying and Freeze Drying Technique on the Antioxidant Properties of Blueberry (*Vaccinium myrtillus*) Juice Polyphenolic Compounds. Pol. J. Food Nutr. Sci..

[22] Wilkowska A., Ambroziak W., Adamiec J., Czyżowska A. (2017). Preservation of Antioxidant Activity and Polyphenols in Chokeberry Juice and Wine with the Use of Microencapsulation. J. Food Process. Preserv..

[23] Escobar-Avello D., Avendaño-Godoy J., Santos J., Lozano-Castellón J., Mardones C., von Baer D., Luengo J., Lamuela-Raventós R.M., Vallverdú-Queralt A., Gómez-Gaete C. (2021). Encapsulation of Phenolic Compounds from a Grape Cane Pilot-Plant Extract in Hydroxypropyl Beta-Cyclodextrin and Maltodextrin by Spray Drying. Antioxidants.

[24] Vidović S.S., Vladić J.Z., Vaštag Ž.G., Zeković Z.P., Popović L.M. (2014). Maltodextrin as a Carrier of Health Benefit Compounds in Satureja Montana Dry Powder Extract Obtained by Spray Drying Technique. Powder Technol..

[25] Caliskan G., Dirim S.N. (2016). The Effect of Different Drying Processes and the Amounts of Maltodextrin Addition on the Powder Properties of Sumac Extract Powders. Powder Technol..

[26] Cai Y.Z., Corke H. (2000). Production and Properties of Spray-Dried Amaranthus Betacyanin Pigments. J. Food Sci..

[27] Goula A.M., Adamopoulos K.G. (2008). Effect of Maltodextrin Addition during Spray Drying of Tomato Pulp in Dehumidified Air: II. Powder Properties. Dry. Technol..

[28] Waterman P.G., Mole S., Waterman P.G., Mole S. (1994). Analysis of Phenolic Plant Metabolites.

[29] Loizzo M.R., Tundis R., Bonesi M., Menichini F., Mastellone V., Avallone L., Menichini F. (2012). Radical Scavenging, Antioxidant and Metal Chelating Activities of Annona Cherimola Mill. (Cherimoya) Peel and Pulp in Relation to Their Total Phenolic and Total Flavonoid Contents. J. Food Compos. Anal..

[30] Bigović D., Savikin K., Janković T., Menković N., Zdunić G., Stanojković T., Djurić Z. (2011). Antiradical and Cytotoxic Activity of Different Helichrysum Plicatum Flower Extracts. Nat. Prod. Commun..

[31] Vladić J., Nastić N., Janković T., Šavikin K., Menković N., Lončarević I., Vidović S. (2022). Microencapsulation of Sideritis Raeseri Boiss. & Heldr. Subsp. Raeseri Extract Using Spray Drying with Maltodextrin and Whey Protein. Period. Polytech. Chem. Eng..

[32] Amidon G.E., Houghton M.E. (1995). The Effect of Moisture on the Mechanical and Powder Flow Properties of Microcrystalline Cellulose. Pharm. Res..

[33] Khelissa S., Gharsallaoui A., Fadel A., Barras A., Jama C., Jbilou F., Chihib N.-E. (2021). Microencapsulation of Benzalkonium Chloride Enhanced Its Antibacterial and Antibiofilm Activities against Listeria Monocytogenes and Escherichia Coli. J. Appl. Microbiol..

[34] Crouter A., Briens L. (2014). The Effect of Moisture on the Flowability of Pharmaceutical Excipients. AAPS PharmSciTech.

[35] Hundre S.Y., Karthik P., Anandharamakrishnan C. (2015). Effect of Whey Protein Isolate and β-Cyclodextrin Wall Systems on Stability of Microencapsulated Vanillin by Spray–Freeze Drying Method. Food Chem..

[36] Pasrija D., Ezhilarasi P.N., Indrani D., Anandharamakrishnan C. (2015). Microencapsulation of Green Tea Polyphenols and Its Effect on Incorporated Bread Quality. LWT-Food Sci. Technol..

[37] Sharifi F., Otte A., Yoon G., Park K. (2020). Continuous In-Line Homogenization Process for Scale-up Production of Naltrexone-Loaded PLGA Microparticles. J. Control. Release.

[38] Kamimura J.A., Santos E.H., Hill L.E., Gomes C.L. (2014). Antimicrobial and Antioxidant Activities of Carvacrol Microencapsulated in Hydroxypropyl-Beta-Cyclodextrin. LWT-Food Sci. Technol..

[39] Tontul I., Topuz A. (2017). Spray-Drying of Fruit and Vegetable Juices: Effect of Drying Conditions on the Product Yield and Physical Properties. Trends Food Sci. Technol..

[40] Zigoneanu I.G., Astete C.E., Sabliov C.M. (2008). Nanoparticles with Entrapped α-Tocopherol: Synthesis, Characterization, and Controlled Release. Nanotechnology.

[41] Sánchez-Hernández E., Álvarez-Martínez J., González-García V., Casanova-Gascón J., Martín-Gil J., Martín-Ramos P. (2023). *Helichrysum stoechas* (L.) Moench Inflorescence Extract for Tomato Disease Management. Molecules.

[42] Martins M.S., Nascimento M.H., Barbosa L.L., Campos L.C.G., Singh M.N., Martin F.L., Romão W., Filgueiras P.R., Barauna V.G. (2022). Detection and Quantification Using ATR-FTIR Spectroscopy of Whey Protein Concentrate Adulteration with Wheat Flour. LWT.

[43] Seo E.-J., Min S.-G., Choi M.-J. (2010). Release Characteristics of Freeze-Dried Eugenol Encapsulated with β -Cyclodextrin by Molecular Inclusion Method. J. Microencapsul..

[44] Cassol L., Noreña C.P.Z. (2021). Microencapsulation and Accelerated Stability Testing of Bioactive Compounds of Hibiscus Sabdariffa. J. Food Meas. Charact..

[45] Jeff Schwegman J., Carpenter J.F., Nail S.L. (2007). Infrared Microscopy for in Situ Measurement of Protein Secondary Structure during Freezing and Freeze-Drying. J. Pharm. Sci..

[46] Izutsu K., Aoyagi N., Kojima S. (2004). Protection of Protein Secondary Structure by Saccharides of Different Molecular Weights during Freeze-Drying. Chem. Pharm. Bull..

[47] Jovanović A.A., Lević S.M., Pavlović V.B., Marković S.B., Pjanović R.V., Đorđević V.B., Nedović V., Bugarski B.M. (2021). Freeze vs. Spray Drying for Dry Wild Thyme (*Thymus serpyllum* L.) Extract Formulations: The Impact of Gelatin as a Coating Material. Molecules.

[48] Loftsson T., Másson M., Brewster M.E. (2004). Self-Association of Cyclodextrins and Cyclodextrin Complexes. J. Pharm. Sci..

[49] Pinho E., Grootveld M., Soares G., Henriques M. (2014). Cyclodextrins as Encapsulation Agents for Plant Bioactive Compounds. Carbohydr. Polym..

[50] Şahin-Nadeem H., Dinçer C., Torun M., Topuz A., Özdemir F. (2013). Influence of Inlet Air Temperature and Carrier Material on the Production of Instant Soluble Sage (Salvia Fruticosa Miller) by Spray Drying. LWT-Food Sci. Technol..

[51] Vladić J., Ambrus R., Szabó-Révész P., Vasić A., Cvejin A., Pavlić B., Vidović S. (2016). Recycling of Filter Tea Industry By-Products: Production of A. Millefolium Powder Using Spray Drying Technique. Ind. Crops Prod..

[52] Mari A., Napolitano A., Masullo M., Pizza C., Piacente S. (2014). Identification and Quantitative Determination of the Polar Constituents in Helichrysum Italicum Flowers and Derived Food Supplements. J. Pharm. Biomed. Anal..

[53] Albayrak S., Aksoy A., Sağdiç O., Budak Ü. (2010). Phenolic Compounds and Antioxidant and Antimicrobial Properties of Helichrysum Species Collected from Eastern Anatolia, Turkey. Turk. J. Biol..

