# Supplementary material for: Development of Cyclodextrin-Based Mono and Dual Encapsulated Powders by Spray Drying for Successful Preservation of Everlasting Flower Extract
Source: Pharmaceutics. 2024 Jun 27;16(7):861. doi: 10.3390/pharmaceutics16070861 (PMC11279902; doi:10.3390/pharmaceutics16070861)

Supplementary material

Table S1. Hygroscopicity of spray-dried *helichrysum* extract (SHE) and microencapsulates

Samples	Day 1	Day 3	Day 4	Day 6
SHE	2.07 ± 0.01 f	2.27 ± 0.05 ef	4.45 ± 0.12 de	5.22 ± 0.09 d
SHE+MD	2.19 ± 0.00 e	2.34 ± 0.06 e	4.74 ± 0.34 cd	5.54 ± 0.10 c
SHE+WP	2.85 ± 0.00 a	2.98 ± 0.01 a	5.61 ± 0.06 a	6.60 ± 0.03 a
SHE+BCD	2.72 ± 0.02 b	2.87 ± 0.00 b	5.30 ± 0.04 ab	5.87 ± 0.05 b
SHE+HPBCD	2.51 ± 0.07 c	2.62 ± 0.05 c	5.22 ± 0.29 ab	5.58 ± 0.03 c
SHE+MD+BCD	2.42 ± 0.05 cd	2.44 ± 0.02 d	4.33 ± 0.04 de	4.66 ± 0.06 f
SHE+WP+BCD	1.83 ± 0.01 g	2.01 ± 0.01 g	4.05 ± 0.02 e	4.20 ± 0.01 g
SHE+MD+HPBCD	2.01 ± 0.03 f	2.21 ± 0.02 f	4.34 ± 0.08 de	4.96 ± 0.08 e
SHE+WP+HPBCD	2.40 ± 0.05 d	2.62 ± 0.00 c	4.97 ± 0.09 bc	5.54 ± 0.00 c

Means followed by different letters are significantly different according to the post hoc Duncan's test al level $p \leq 0.05$ SHE-spray-dried *Helichrysum* extract, MD-maltodextrin, WP-whey protein, BCD- β-cyclodextrin, HPBCD-hydroxypropyl-β-cyclodextrin.

Figure S1. The high-performance liquid chromatography (HPLC) spectrum of the *H. plicatum* extract powder: 1, 2, 3- glycosylated naringenin derivatives, 4- isoquercitrin, 5- kaempferol-3-*O*-glucoside, 6- apigenin-7-*O*-glucoside, 7- naringenin, 8- apigenin, 9- kaempferol

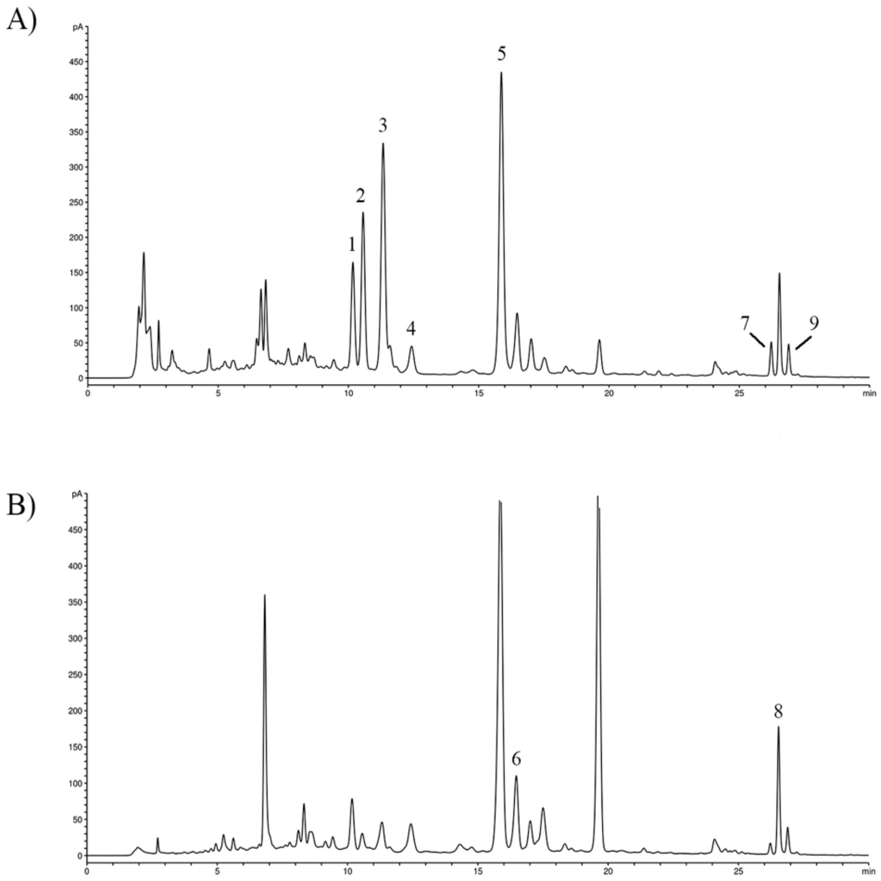

Supplement: Supplementary file 1 [file pharmaceutics-16-00861-s001.zip › pharmaceutics-3054638-supplementary.pdf]
